# Protective Effects of Liposomal Curcumin on Oxidative Stress/Antioxidant Imbalance, Metalloproteinases 2 and -9, Histological Changes and Renal Function in Experimental Nephrotoxicity Induced by Gentamicin

**DOI:** 10.3390/antiox10020325

**Published:** 2021-02-22

**Authors:** Adriana Elena Bulboacă, Alina Porfire, Sorana D. Bolboacă, Cristina Ariadna Nicula, Dana Gabriela Feștilă, Alexandra Roman, Ruxandra Mioara Râjnoveanu, Armand Râjnoveanu, Gabriela Dogaru, Paul-Mihai Boarescu, Vasile Rus, Corneliu Angelo Bulboacă, Alexandra Ina Bulboacă, Ioana Stănescu

**Affiliations:** 1 Department of Pathophysiology, Iuliu Hațieganu University of Medicine and Pharmacy, 400012 Cluj-Napoca, Romania; adriana.bulboaca@umfcluj.ro (A.E.B.); boarescu.paul@umfcluj.ro (P.-M.B.); 2 Department of Pharmaceutical Technology and Biopharmaceutics, Iuliu Hațieganu University of Medicine and Pharmacy, 400012 Cluj-Napoca, Romania; alinatuns@yahoo.com; 3 Department of Medical Informatics and Biostatistics, Iuliu Hațieganu University of Medicine and Pharmacy, 400349 Cluj-Napoca, Romania; 4 Department of Ophthalmology, Iuliu Hațieganu University of Medicine and Pharmacy, 400012 Cluj-Napoca, Romania; cristina.nicula@umfcluj.ro; 5 Department of Orthodontics, Iuliu Hațieganu University of Medicine and Pharmacy, 400349 Cluj-Napoca, Romania; dana.festila@gmail.com; 6 Department of Periodontology, Iuliu Hațieganu University of Medicine and Pharmacy, 400012 Cluj-Napoca, Romania; veve_alexandra@yahoo.com; 7 Department of Pneumology, Iuliu Haţieganu University of Medicine and Pharmacy, 400371 Cluj-Napoca, Romania; andra_redro@yahoo.com; 8 Department of Occupational Medicine, Iuliu Haţieganu University of Medicine and Pharmacy, 400349 Cluj-Napoca, Romania; armand.rajnoveanu@umfcluj.ro; 9 Department of Physical Medicine and Rehabilitation, Iuliu Haţieganu University of Medicine and Pharmacy, 400347 Cluj-Napoca, Romania; dogarugabrielaumf@gmail.com; 10 Department of Cell Biology, Histology and Embryology, University of Agricultural Sciences and Veterinary Medicine, 400375 Cluj-Napoca, Romania; vasile.rus@usamvcluj.ro; 11 Department of Neurology, Iuliu Haţieganu University of Medicine and Pharmacy, 400012 Cluj-Napoca, Romania; angelo.bulboaca@yahoo.com (C.A.B.); ioana.stanescu.umfcluj@gmail.com (I.S.); 12 Faculty of Medicine, Iuliu Haţieganu University of Medicine and Pharmacy, 400349 Cluj-Napoca, Romania; bulboaca.alexandra@yahoo.com

**Keywords:** Gentamicin induced nephrotoxicity, curcumin, oxidative stress

## Abstract

Background: Our study aimed to assess the efficiency of Curcumin nanoformulation (LCC) on experimental nephrotoxicity induced by Gentamicin in rats. Methods: Six groups of seven rats were used: C—(control group) received saline solution i.p. (i.p. = intraperitoneal), G—gentamicin (G, 80 mg/kg body weight (b.w.)), GCC1 and GCC2—with G and CC solution (single dose of 10 mg/kg b.w.-CC1, or 20 mg/kg b.w.-CC2), GLCC1 (10 mg/kg b.w.) and GLCC2 (20 mg/kg b.w.) with G and LCC administration. Oxidative stress parameters (NOx = nitric oxide, MDA = malondialdehyde, TOS = total oxidative stress), antioxidant parameters (CAT = catalase, TAC = total antioxidant capacity), matrix metalloproteinases (MMP-2 and MMP-9), and renal function parameters (creatinine, blood urea nitrogen, and urea) were measured. Kidneys histopathologic examination was made for each group. Results: Pretreatment with CC and LCC in both doses had significantly alleviating effects on assessed parameters (NOx, MDA, TOS, CAT, TAC, MMP-2, and -9) as compared with the untreated group (*p* < 0.006). Histopathological aspect and renal function were significantly improved in CC and LCC groups. Liposomal formulation (LCC) showed higher efficiency on all examined parameters compared to CC (*p* < 0.006). Conclusions: Our results demonstrated improving renal function and kidney cytoarchitecture, oxidative stress/antioxidant/balance, and MMPs plasma concentrations with better dose-related efficacity of LCC than CC.

## 1. Introduction

Aminoglycoside antibiotics are widely used for gram-negative microorganism infections. This class comprises Gentamicin, Tobramycin, and Amikacin, their bactericidal activity being concentration-dependent [1]. One of the most important utilities of amino glycosides is represented by their efficacy on nosocomial infections, a serious public health problem. Various antibiotic therapies proved to be useful in nosocomial infections treatment. In the group of aminoglycosides (antibiotics used to treat severe nosocomial gram-negative infections), Gentamicin was proved to be an effective treatment for nosocomial infections [2]. Aminoglycosides exert their antibacterial activity due to impeding bacterial synthesis of proteins by increasing cell’s membrane permeability [3]. The result consists in bacteria membrane instability, leading finally to microorganism death [4].

The use of gentamicin, in parenteral administration, has shown significant better infection outcomes involving some gram-negative bacteria such as *Escherichia coli*, *Klebsiella pneumoniae*, *Pseudomonas aeruginosa*, and was also efficient against some other strains such as Neisseria [5,6,7]. Gentamicin also proved to be efficient in bacterial septicemia, meningitis, peritonitis, endocarditis, pneumonia, urinary tract infections, or odontogenic infections [8]. Local antimicrobial resistance and side effects of Gentamicin’s clinical use are important to consider before Gentamicin treatment initiation. A combination of Gentamicin with another antibiotic, such as beta-lactams is used to facilitate the effect on gram-negative bacteria [9]. Liposomal formulation of Gentamicin could improve the efficiency due to better penetration of nanoparticles into the target cells [2]. Despite its high effectiveness, nephrotoxicity must be considered since renal failure is reported in 10–20% of cases during therapy [10]. Different mechanisms of Gentamicin induced nephrotoxicity have been studied, pointing on inflammatory reaction and oxidative stress-induced renal proximal tubular epithelial cells (RPTECs) apoptosis [11].

### 1.1. Oxidative and Nitrosative Stress in Renal Injury

Amplification of oxidative and nitrosative stress in renal injury induced by various etiologic factors holds a key role in the pathogenesis of renal lesion’s evolution and consequences [12]. Many enzymatic processes in mitochondria contribute to reactive oxygen species (ROS) generation, high reactive molecules, which in pathological conditions can produce significant lesions due to oxidation of proteins, lipids, and DNA (deoxyribonucleic acid). The kidney tissue is rich in mitochondria, and as a consequence, a disrupted balance of kidney cellular metabolic processes can result in ROS production [13]. The most important ROS are represented by superoxide (O_2_^−^), hydrogen peroxide (H_2_O_2_), nitric oxide, peroxynitrite (ONOO^−^), and hypochlorite (ClO^−^). Superoxide anion (O_2_^−^) reduces oxygen by nicotinamide adenine dinucleotide phosphate (NADPH) oxidase action. Superoxide anion is reacting with nitric oxide (NO), resulting in peroxynitrite (nitrosative stress). Peroxynitrite is a high oxidative molecule leading to proteins and DNA damages. On the other hand, superoxide anion leads to hydrogen peroxide (H_2_O_2_) formation by the superoxide dismutase (SOD) action. Hydrogen peroxide may partially be reduced to hydroxide ion and hydroxyl radical (OH^−^) or completely reduced to H_2_O [14,15]. These molecules exert an important signaling function in normal conditions. Their harmful effect is conditioned by the production rate, quantity and the antioxidant systems’ ability to reduce their activity [16]. Renal dysfunction can result from amplification of oxidative/nitrosative stress due to various triggers that exert their effect by augmentation of these reactive molecules’ production. The pathological conditions can be associated with toxic chemicals (including drugs such as Gentamicin), diabetic nephropathy, glomerulonephritis, diabetic neuropathy, and renal ischemia by various causes [12,13]. In some instances, oxidative and nitrosative molecules constitute signal molecules for triggering the inflammatory process [17,18].

### 1.2. Matrix Metalloproteinases in Renal Injury

One of the most important molecules contributing to renal failure is represented by metalloproteinases (MMPs), a group of zinc-dependent proteinases responsible for extracellular matrix remodeling [19]. MMPs (also called matrixins) are subdivided into collagenases, gelatinases, stromelysisns, matrilysins, membrane type MMPs, metalloelastase, enamelysin, and other MMPs types. They contribute to inflammation regulation, cell proliferation, angiogenesis (in normal and pathological conditions), and apoptosis [20]. The balance of MMPs activity is provided by their natural inhibitors, tissue inhibitors of metalloproteinases (TIMPs), that are designed to limit the actions of MMPs [21]. Renal injury resulting from various kidney disorders is associated with MMPs dysregulation and their natural inhibitors (TIMP) dysfunction. Hereditary kidney diseases, toxic kidney injury, glomerulonephritis, diabetic nephropathy, or chronic allograft nephropathy can be associated with MMPs/TIMP dysregulation [20,21,22]. Most of the researches are focused on gelatinase A (MMP-2) and gelatinase B (MMP-9) plasma concentration [21]. They are involved in the breakdown of the glomerular basement membrane disruption and renal inflammation and fibrosis in accelerating renal disease progression [23]. MMP-9 can serve as a biomarker for acute kidney injury [23]. Both MMP-2 and MMP-9 are increased perivascular in acute kidney injury [24], and systemic effects were also proved by high levels of MMP-2 and -9 in extrarenal circulation in chronic renal diseases [25]. MMP-2 has been demonstrated to facilitate the epithelial-mesenchymal transition (the epithelium’s conversion to a fibroblastic or myofibroblastic phenotype) [26]. Moreover, one of the most important MMPs activators is represented by oxidative and nitrosative stress [27]. These evidences show that renal injury results from the interplay between various molecules that can act in a cascade, and the attenuation of one contributor can significantly decrease the lesions. 

Nutraceutical compound curcumin (CC) has been demonstrated to affect oxidative stress, nitrosative stress, and inflammation reduction in various pathological conditions [28,29,30,31]. Due to low availability, different pharmaceutical formulas have been studied, showing their ability to enhance these beneficial effects of CC. One of the most efficient CC formulations is represented liposomal Curcumin nanoformulation, which proved antioxidative and anti-inflammatory effects [32]. Liposomes are a new formulation consisting of a bilayer lipid vesicle that can encapsulate various therapeutic molecules. Liposomes mimic the cell’s membranes’ model to carry the bioactive molecules and increase their efficacy [33]. Liposomal Curcumin (LCC) presents bioavailability advantages due to its ability to protect and deliver the curcumin molecules to the target tissues. The most used administration route is the parenteral administration, as the oral administration route is associated with gastrointestinal degradation of liposomes and low bioavailability [34].

### 1.3. Protective Effects of Curcumin against Gentamicin-Induced Nephrotoxicity

The protective effect of Curcumin on Gentamicin-induced nephrotoxicity (GMN) was in the attention of researchers all over the world. Curcumin oral solution in a concentration of 200 mg/kg/day increases the glutathione (GSH) concentration and superoxide dismutase (SOD) on GMN and significantly reduces the gentamicin (GM) concentration (by 39%, *p* < 0.05) in the renal cortex [35]. Farombi and Ekor proved the effects of Curcumin (administrated orally in a dose of 200 mg/kg/day) on the antioxidative damage induced by GM, attenuating the reduction of catalase (CAT) by 31%, GSHPx (gutathione peroxidase) by 55%, level of GSH by 74%, the increase in plasma malondialdehyde (MDA) by 57%, and kidney MDA by 62% [36]. Manikandan et al. [37] demonstrated the enhanced antioxidants (SOD, CAT, GSHPx, and GSH) and decreases of nitric oxide synthase (iNOS) and nuclear factor-κB (NF-κB) of Curcumin (oral administration, 200mg/kg b.w., b.w. = body weight). The Curcumin solution’s (orally administrated) alleviation effect on altered biochemical and histopathological features on GMN was also demonstrated by other authors [38,39,40]. The synergic effects of Curcumin in GMN with different compounds such as thymoquinone [38], metformin [40], were also reported in the scientific literature. The protective effect of Curcumin on rats was also demonstrated for nephrotoxicity induced by other methods (zinc [41], cyclosporine [41], colistin [42], or cisplatin [43]). 

The previously reported effects of Curcumin on induced nephrotoxicity are mainly limited to the Curcumin solution, which is known to have low bioavailability. The scientific literature is scarce when it comes to other forms of Curcumin formulation. El-Gizawy et al. showed the effect of Curcumin nanoparticles (CUR NPs) in a dose of 50 mg/kg b.w./day [44]. The CUR NPs improved urea (15.13%), creatinine (6.74%), and uric acid (11.11%) levels as compared to controls, but the results did not reach statistical significance. The CUR NPs significantly alleviated creatinine (*p* = 0.003) and uric acid (*p* < 0.001) in the cisplatin induced nephrotoxicity [44]. 

### 1.4. Study Aim

Our study aimed to evaluate the comparative effect of conventional Curcumin (CC) with Curcumin nanoparticles (liposomal Curcumin (LCC)) on an experimental model of Gentamicin induced nephrotoxicity in rats by assessing oxidative stress parameters and metalloproteinases. The effects of CC and LCC were evaluated for two doses in order to elucidate the existence of a dose-dependent effect.

## 2. Materials and Methods

### 2.1. Experimental Design

**Figure 1:**
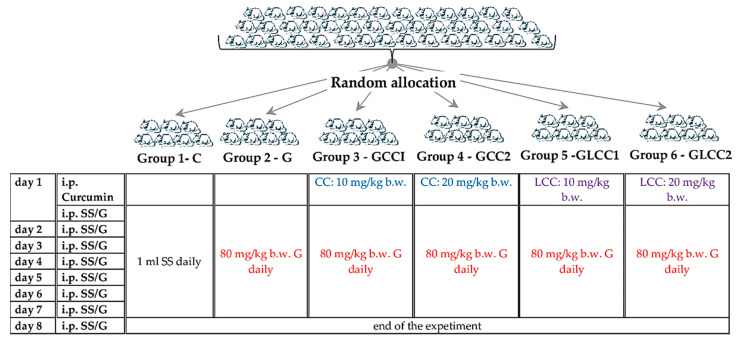
Experimental design schema. SS = saline solution, G = Gentamicin. curcumin solution (CC) and liposomal Curcumin (LCC) solutions were administrated as single dose 30 min before the first dose of Gentamicin, in the first day of the experiment. i.p. = intraperitoneal; b.w. = body weight.

The animals were procured from the Centre for Experimental Medicine and Practical Skills and randomly divided into six groups, seven animals for each group. Wistar–Bratislava albino male rats (weighing 200–250 g) were used for this experimental study. The animals were kept in polypropylene cages at constant temperature (24 ± 2 °C), 60 ± 5% humidity, and light-dark regime. The animals received unrestricted access to food (standard pellets from Cantacuzino Institute, Bucharest, Romania) and water. The curcumin solution (CC) and liposomal Curcumin (LCC) were used for treated groups. The flow of the experiment is presented in Figure 1.

### 2.2. Chemicals

The materials were purchased as follows: Curcumin (CC), cholesterol, and Gentamicin from Sigma-Aldrich Co (St Louis, MO, USA), 1,2-Dipalmitoyl-sn-glycero-3-phosphocholine (DPPC, ≥99% [TP-PC]) and *N*-(-2000)-1,2-distearoyl-sn-glycero-3phosphoethanolamine (PEG-2000-DSPE, ≥98% [HPLC]) sodium salt from Lipoid GmbH (Ludwigshafen, Germany). Metalloproteinases assessment was made with a kit purchased from R&D Systems Quantikine (McKinley Place NE, Minneapolis, USA). All other chemicals were of analytical grade.

Liposomal Curcumin was represented by PEG-ylated liposomes, prepared according to the method previously described [45,46]. Briefly, the liposomal dispersion was obtained by hydration of a lipid film containing the lipid mixture (DPPC:PEG-2000-DSPE:CHO in a molar ratio of 9.5:0.5:1) and Curcumin (5 mg/mL) and was extruded afterwards through polycarbonate membranes using LiposoFast LF-50 extruder (Avestin Europe GmbH, Mannheim, Germany). The extruded dispersion was characterized in terms of Curcumin concentration (by HPLC method [46]), particle size, and size distribution (by dynamic light scattering using a Zetasizer Nano ZS from Malvern Instruments, Malvern, UK). The resulted dispersion contained around 4 mg/mL Curcumin, had a particle size around 140 nm, and the particle size distribution was narrow (polydispersity index lower 0.1), having thus a quality profile appropriate for intravenous administration. Curcumin solution (CC, 20% *v*/*v* ethanol) of the same concentration as in liposomes was obtained by dissolution in 96% (*v*/*v*) ethanol and subsequent dilution with saline.

### 2.3. Markers Measurements

On the 8th day, blood samples were collected for the estimation of oxidative stress parameters such as nitric oxide (NOx), malondialdehyde (MDA), total oxidative stress (TOS), and antioxidant parameters such as catalase (CAT) and total antioxidant capacity (TAC). Matrix metalloproteinases (MMP-2 and MMP-9), renal function (creatinine, blood urea nitrogen (BUN), and urea) were also assessed. At the end of the experiment, all the animals were euthanized after general anesthesia (ketamine—5 mg/kg body weight, i.p. route) [47] by cervical dislocation, after the blood sample collection from retro-orbital plexus. 

A Jasco V-350 UV-VIS spectrophotometer (Jasco International Co, Ltd., Tokyo, Japan) was used for all measurements. The oxidative stress intensity (NOx, MDA, and TOS) and antioxidant capacity of plasma (CAT and TAC) parameters were measured by the methods described somewhere else [48]. Metalloproteinases assessment was made with a Stat Fax 303 ELISA reader (Quantikine, McKinley Place NE, MN, USA), using a rat ELISA kit (Boster Biological Technology, Pleasanton, CA, USA). Renal function parameters assessment was made by colorimetric methods [49,50], and the reagents were purchased from Spinreact (Girona, Spain).

### 2.4. Histological Evaluation

For histological examination, fragments of longitudinal slices with a thickness of 4 mm were collected from the kidneys and fixed by immersion in Stieve mixture for 24 h. At the end of the fixation period, the samples were processed histologically by inclusion in paraffin, sectioned at 5 µm, and the Goldner trichrome stain was used. The microscopic preparations were examined under an optical microscope (Olympus BX40, Hamburg, Germany), and photographs were taken (Olympus E-330, Hamburg, Germany) and digitally processed (Adobe Photoshop CS2, San Jose, CA, USA).

### 2.5. Statistical Analysis

Data are expressed as mean and standard deviation. The differences in the evaluated parameters were investigated using the Kruskal–Wallis ANOVA test. The differences between groups regarding oxidative stress markers, metalloproteinases and renal function parameters levels were assessed with the Mann–Whitney test. In figures, the distribution of investigated parameters in groups was plotted as individual values (circles) and the median (line) as recommended by Weissgerber et al. [51]. The significance level of 5% was adjusted by the number of investigated groups whenever more than two groups were compared.

## 3. Results

No rat died during the experiment, therefore, statistical analysis was performed on all seven rats in each group.

### 3.1. Oxidant/Antioxidant Balance

**Figure 2:**
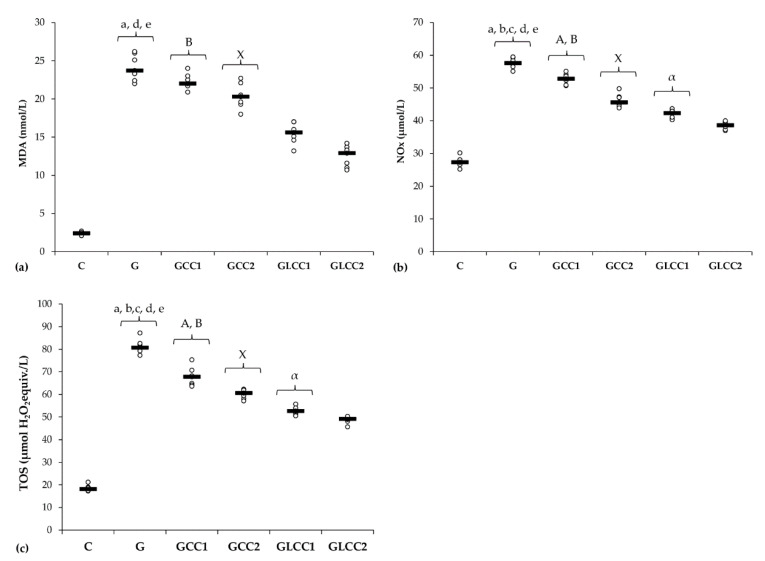
Variation by groups of serum oxidative stress intensity: (**a**) MDA (malondialdehyde), (**b**) NOx (nitric oxide), (**c**) TOS (total oxidative status) by groups. Notes: The circles represent the individual values, and the horizontal line is given by the median. Abbreviations: C, Control; G, Gentamicin; GCC1, Gentamicin and CC1 solution; GCC2, Gentamicin and CC2 solution; GLCC1, Gentamicin and LCC1 solution; GLCC2, Gentamicin and LCC2 solution; The letter codes correspond to the *p*-values < 0.006: ^a^ G compared to C; ^b^ G compared to GCC1; ^c^ G compared to GCC2; ^d^ G compared to GLCC1; ^e^ G compared to GLCC2; ^A^ GCC1 compared to GCC2; ^B^ GCC1 compared to GLCC1; ^X^ GCC2 compared to GLCC2; ^α^ GLCC1 compared to GLCC2.

**Figure 3:**
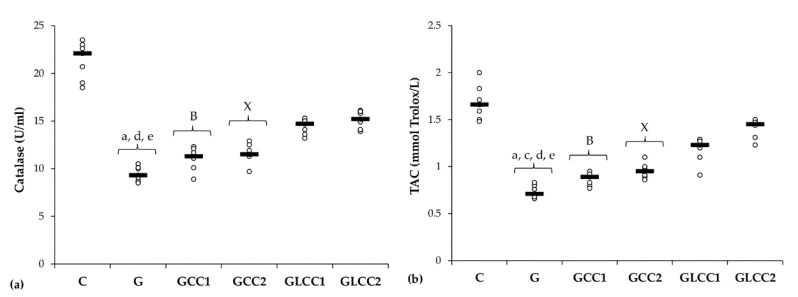
Variation by groups of serum antioxidant capacity: (**a**) Catalase and (**b**) TAC (total antioxidant capacity) by groups. Notes: The circles represent the individual values, and the horizontal line is given by the median. Abbreviations: C, Control; G, Gentamicin; GCC1, Gentamicin and CC1 solution; GCC2, Gentamicin and CC2 solution; GLCC1, Gentamicin and LCC1 solution; GLCC2, Gentamicin and LCC2 solution; The letter codes correspond to the *p*-values < 0.006: ^a^ G compared to C; ^c^ G compared to GCC2; ^d^ G compared to GLCC1; ^e^ G compared to GLCC2; ^B^ GCC1 compared to GLCC1; ^X^ GCC2 compared to GLCC2.

Gentamicin administration increased serum levels of all oxidative stress parameters: MDA, NOx, and TOS (Table 1 and Figure 2) and reduced antioxidant capacity: catalase and TAC (Table 2 and Figure 3).

Conventional curcumin administration reduced serum levels of NOx and TOS (*p* ≤ 0.0026, Table 1, Figure 2) but did not significantly affect the MDA (*p* ≥ 0.0073, Table 1, Figure 2). Elevation of all oxidative stress parameters proved dose-dependent and more effective for LCC than standard curcumin (*p* ≤ 0.0022, Table 1, Figure 2).

Curcumin in lower dose did not significantly influence the serum level of antioxidant capacity parameters (*p* ≥ 0.0104, Table 2, Figure 3), while the highest dose increased only serum level of TAC (*p* = 0.0022, Table 2, Figure 3). Both doses of curcumin nanoparticles enhanced the antioxidant defense by increasing serum level of catalase and TAC (*p* = 0.0022, Table 2, Figure 3). For antioxidant capacity, no statistical differences were found between used doses, neither for conventional Curcumin, nor for liposomal Curcumin (*p* ≥ 0.0087, Table 2, Figure 3).

### 3.2. Matrix Metalloproteinases

**Figure 4:**
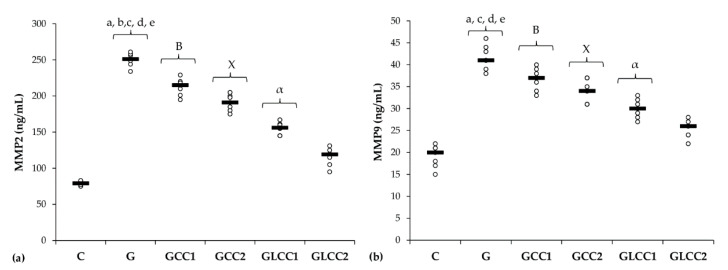
Variation by groups of matrix metalloproteinases: (**a**) MMP-2 (matrix metalloproteinases 2) (**b**) MMP-9 (matrix metalloproteinases 9) by groups. Notes: The circles represent the individual values, and the horizontal line is given by the median. Abbreviations: C, Control; G, Gentamicin; GCC1, Gentamicin and CC1 solution; GCC2, Gentamicin and CC2 solution; GLCC1, Gentamicin and LCC1 solution; GLCC2, Gentamicin and LCC2 solution; The letter codes correspond to the *p*-values < 0.006: ^a^ G compared to C; ^b^ G compared to GCC1; ^c^ G compared to GCC2; ^d^ G compared to GLCC1; ^e^ G compared to GLCC2; ^B^ GCC1 compared to GLCC1; ^X^ GCC2 compared to GLCC2; ^α^ GLCC1 compared to GLCC2.

Serum levels of matrix metalloproteinases (MMP-2 and MMP-9) increased after gentamicin administration. (Table 3 and Figure 4).

The lowest CC dose reduced only serum level of MMP-2 (*p* = 0.0022, Table 3, Figure 4), while the highest dose reduced both MMPs (*p* ≤ 0.0022, Table 3, Figure 4). Best results in reducing MMP-2 and MMP-9 were obtained after administering curcumin nanoparticles in the highest dose (*p* ≤ 0.0022, Table 3, Figure 4).

### 3.3. Renal Function Parameters

**Figure 5:**
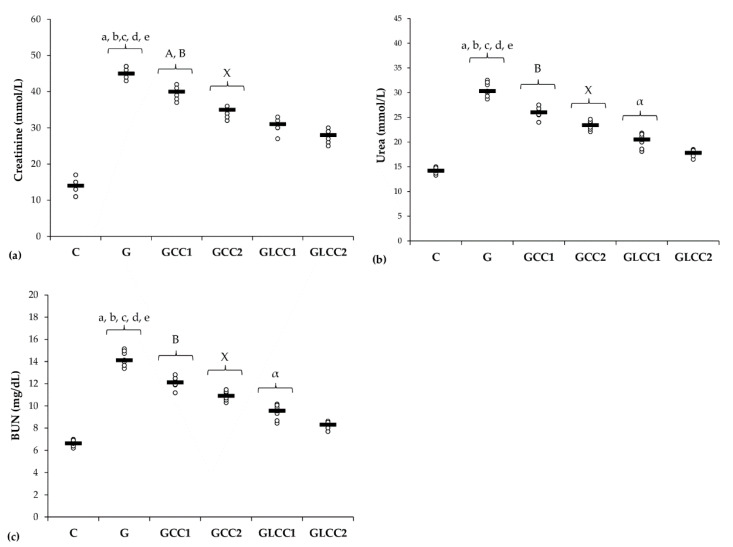
Variation by groups of serum levels of renal function parameters: (**a**) Creatinine, (**b**) Urea, (**c**) (BUN) blood urea nitrogen by groups. Notes: The circles represent the individual values, and the horizontal line is given by the median. Abbreviations: C, Control; G, Gentamicin; GCC1, Gentamicin and CC1 solution; GCC2, Gentamicin and CC2 solution; GLCC1, Gentamicin and LCC1 solution; GLCC2, Gentamicin and LCC2 solution; The letter codes correspond to the *p*-values < 0.006: ^a^ G compared to C; ^b^ G compared to GCC1; ^c^ G compared to GCC2; ^d^ G compared to GLCC1; ^e^ G compared to GLCC2; ^A^ GCC1 compared to GCC2; ^B^ GCC1 compared to GLCC1; ^X^ GCC2 compared to GLCC2; ^α^ GLCC1 compared to GLCC2.

Rats from the group with Gentamicin and saline administration had the highest levels of renal function parameters (Table 4, Figure 5).

All doses of standard Curcumin and liposomal Curcumin reduced serum creatinine, urea, and BUN with best results for liposomal Curcumin (*p* ≤ 0.0049, Table 4, Figure 5).

No statistical differences were found between doses of standard Curcumin for urea and BUN, neither for liposomal Curcumin for creatinine (*p* ≥ 0.006, Table 4, Figure 5).

### 3.4. Histopathological Changes

**Figure 6:**
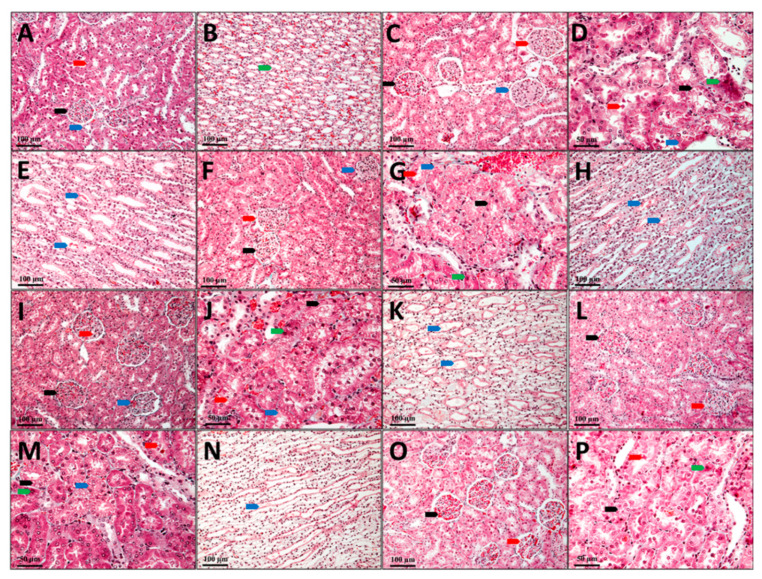
Kidney, Goldner trichrome coloration; (**A**,**B**) C, Control; (**C**–**E**) G, Gentamicin; (**F**–**H**) GCC1, Gentamicin and CC1 solution; (**I**–**K**) GCC2, Gentamicin and CC2 solution; (**L**–**N**) GLCC1, Gentamicin and LCC1 solution; (**O**,**P**) GLCC2, Gentamicin and LCC2 solution; (**A**), black arrow—renal corpuscle, red arrow—proximal tube, blue arrow—distal tube; (**B**), green arrow–collector tube; (**C**), black arrow—renal corpuscle with capillary ectasia, red arrow—renal corpuscle with glomerular edema, blue arrow—renal corpuscle with mesangial hyperplasia; (**D**), black arrow—nephrocytes with vacuolar degeneration, red arrow—cellular detritus in the lumen of the nephron, blue arrow—intracytolasmatic hyaline, green arrow—dead nephrocytes; (**E**), blue arrow—collector tube with protein precipitates; (**F**), black arrow—renal corpuscle with capillary ectasia, red arrow—renal corpuscle with glomerular edema, blue arrow—renal corpuscle with mesangial hyperplasia; (**G**), black arrow—nephrocytes with vacuolar degeneration, red arrow—cellular detritus in the lumen of the nephron, blue arrow—intracytolasmatic hyaline, green arrow—dead nephrocytes; (**H**), blue arrow—collector tube with protein precipitates; (**I**), black arrow—renal corpuscle with capillary ectasia, red arrow—renal corpuscle with glomerular edema, blue arrow—renal corpuscle with mesangial hyperplasia; (**J**), black arrow—nephrocytes with granulo-vacuolar degeneration, red arrow—cellular detritus in the nephron lumen, blue arrow—intracytolasmatic hyalinosis, green arrow—dead nephrocytes; (**K**), blue arrow—collector tube with protein precipitates; (**L**), black arrow—nephrocytes with vacuolar degeneration, red arrow—cellular detritus in the lumen of the nephron; (**M**), black arrow—nephrocytes with vacuolar degeneration, red arrow—cellular detritus in the lumen of the nephron, blue arrow—in-tracytolasmatic hyaline, green arrow—dead nephrocytes; (**N**), blue arrow—collector tube with protein precipitates; (**O**), black arrow—renal corpuscle with capillary ectasia, red arrow—renal corpuscle with glomerular edema; (**P**), black arrow—nephrocytes with granular degeneration, red arrow—cellular detritus in the nephron lumen, green arrow—dead nephrocytes.

In the control group (C), the kidney’s cytoarchitecture is normal, and no aspects that would suggest renal dysfunction were observed (Figure 6A,B).

Most nephrons in the group that received only Gentamicin (G) show changes in renal corpuscles and tubes, with different damage degrees. Ectasia of the capillaries in the glomerulus with narrowing of the Bowman space were observed in some renal corpuscles. There is pronounced edema in the mesangium in other renal corpuscles, while the number of red blood cells is very small in the glomerular capillaries. Some renal corpuscles present even a proliferation of the mesangium. In these renal corpuscles, the urinary space is also very narrow, and the parietal leaf of the Bowman capsule is thickened. In most renal corpuscles, protein granules with a fine granular appearance are found in the Bowman capsule (Figure 6C). Structural changes with differences from one tube to another are visible in nephron tubes. Generally, the proximal tubules were more affected than the distal tubules. The convoluted segment of the proximal tubes was more affected than the straight segment (at the Ferrein pyramid level). The presence of a fine granular protein precipitate and different cellular detritus quantities were observed in the proximal tubes’ lumen. Degenerative aspects of different degrees are visible in many nephrocytes. The presence of hyaline inclusions varying from very small to large is seen in some of the nephrocytes from the proximal convoluted tubules. Other nephrocytes have granular, granulo-vacuolar, and vacuolar degeneration.

Dead cells are seen in small numbers (Figure 6D). In the distal tubules, most nephrocytes have a more reduced degree of damage than those in the proximal tubules. In the distal tubules, neither dead nephrocytes nor nephrocytes with intracytoplasmic hyalinosis were observed. Only a few nephrocytes with vacuolar degeneration can be observed in this area. Most nephrocytes have a slight granular degeneration. The lumen of the distal tubes has a reduced amount of protein. Protein precipitates in small quantities are found in the collecting tubes at the Malpighi’s pyramid, but they do not form hyaline thrombi that obliterate the tube’s lumen (Figure 6E).

The histological aspects of the kidneys of the animals from GCC1 group are comparable to those described in the G group; still, pathological changes present in this group are not so well expressed. The renal corpuscles present in vascular ectasia, edema, mesangial hyperplasia, and glomerular ectasia are predominant on the sections from the kidneys’ surface. There are some glomeruli in which the edema of the mesangium is intense. In contrast, the number of glomeruli in which the mesangium profiling and thickening process of the Bowman capsule’s parietal sheet is present is very reduced (Figure 6F). In the nephron tubes, the nephrocyte damage degree is slightly reduced, as the number of dead nephrocytes is lower, and reduced areas of vacuolar degeneration can be seen. Instead, most nephrocytes show aspects of intracytoplasmic hyalinosis and granular or granulo-vacuolar degeneration (Figure 6G). The number of nephrocytes with vacuolar degeneration is minimal in the distal tubules, and those with granular or granulo-vacuolar degeneration are significantly less than in G group. Protein precipitates and cellular detritus are also present in the tubes’ lumen, but the amount seems to be slightly smaller than in G group (Figure 6H).

In animals from the GCC2 group, the histological aspects are comparable to those in the GCC1 group. Only that damage degree and the number of tubes with advanced irreversible changes such as vacuolar degeneration are lower, and vascular ectasia predominates in the renal corpuscles. Some renal corpuscles with mesangial edema are observed, while those with mesangial hypertrophy are very rare. Small amounts of protein precipitate and cellular detritus are seen in both the Bowman space and the tube lumen (Figure 6I–K).

Animals from the GLCC1 group have histological aspects similar to those described in the previous groups, but pathological changes of the nephrons are not so advanced. In most corpuscles glomerular ectasia is present, while in other glomeruli mesangial edema is present and mesangial hyperplasia is found only in a few glomeruli. The intracytoplasmic hyalinosis and granular or granulo-vacuolar degeneration are predominant in the nephron tubes, and more rarely advanced aspects of vacuolar degeneration are seen. Protein precipitates, as well as cellular detritus, are visible in the lumen of the Bowman space as well as in the lumen of the tubes (Figure 6L–N).

On the examined sections of the animals’ kidneys from the GLCC2 group, the lesions are not as advanced and do not seem to include most of the nephrons as those from GLCC1 group. A normal aspect of the tissue with normal proximal convoluted tubes and striated appearance of the cytoplasm in the basal half of the cells can be seen in some parts of the sections. Otherwise, the pathological changes are similar to those described above (Figure 6O–P).

## 4. Discussion

### 4.1. Curcumin Influence on Oxidant/Antioxidant Balance in Nephrotoxicity Induced by Gentamicin

This study shows that curcumin administration can improve in a dose-dependent manner plasma oxidative stress parameters/antioxidant capacity, MMP-2 and -9 level, and renal function parameters in nephrotoxicity induced by Gentamicin. Three main mechanisms can explain the nephrotoxicity of Gentamicin: reduced renal blood flow, reduced glomerular filtration, and renal tubular toxicity, all of them associated with renal ischemia and severe renal dysfunction [1]. One of the most important mechanisms associated with kidney dysfunction is represented by oxidative stress augmentation [52]. In our study, the evaluation of oxidative stress parameters (MDA, NOx, and TOS) was suggestive for a better nanocurcumin efficiency. Administration of CC did not improve MDA level concentration (Table 1, Figure 2). On the other hand, NOx and TOS levels were significantly reduced in groups with CC1 and CC2 administration, with better results for CC2 (regarding the NOx) level (Table 1, Figure 2). Liposomal Curcumin (both concentrations, LCC1and LCC2) improved all the oxidative stress parameters (MDA, NOx, TOS), still the level of MDA showed no difference of statistical significance at different doses of LCC administration (Table 1, Figure 2). To describe the mechanism by which these results can be interpreted, we have to consider the differences between administration routes and bioavailability of Curcumin in various formulas, nanocurcumin formula showing to be more efficient. Oral administration of Curcumin limits its bioavailability due to low solubility of Curcumin in water, low intestinal permeability and absorption, instability at physiological pH, and slow cell’s membrane crossing [53,54]. Parenteral administration of Curcumin improved its efficacy by alleviating oxidative stress parameters on experimental induced oxidative stress by nitro-glycerine administration in rats [46]. Kidney concentration of curcumin nanoformulation was increased compared with conventional Curcumin proving a better bioavailability for kidney tissue [55]. Therefore, the pharmacodynamics perspective and renal tissue dispersion of Curcumin can be improved by nanoformulation. Moreover, lipid-based systems, including liposomes for curcumin administration, improve curcumin dissolution and bioavailability, facilitating cell’s membrane crossing [56]. Due to these mechanisms, liposomal Curcumin, by intraperitoneal administration, exerts a better renoprotective effect than curcumin solution. The significant drops of NOx level both in curcumin solution and liposomal curcumin administration, in experimental Gentamicin induced nephrotoxicity, contribute to nitro-oxidative stress reduction by Curcumin, which can alleviate the renal function and histopathological features in all treated groups (Figure 6). The capacity of Curcumin to reduce the elevation of NOx has been previously demonstrated by and the reduction of inducible nitric oxyde synthase, caspase-3 levels, and significant reduction of ROS species production by mitochondria [57]. Improving the mitochondrial function, it reduces the amplitude of intracellular signaling for death triggering [57]. Oxidative and nitrosative stress related to inflammation can be subsequently reduced by this mechanism [58,59]. Amelioration of the oxidative stress (by reducing MDA), inflammation (by reducing TNF-α and ICAM-1 mRNA expression levels), and histopathological changes produced by Gentamicin administration in rats was also proved by other researches for different nutraceutical products such as Malva sylvestris extract [60]. Attenuation of oxidative stress and inflammation produced by Gentamicin nephrotoxicity was reported to improve biomarkers for inflammation such as TNF-α, IL-6, NF-κB, and apoptotic markers (Caspase-3, Bax, and Bcl-2) [61]. Reducing oxidative stress and inflammation is followed by a decrease of autophagy and apoptotic process, with better survival of renal cells to toxic substances induced renal lesions [62]. These reports showed that various nutraceutical products could influence renal toxicity induced by Gentamicin administration in rats.

The dose-related effect observed for NOx level and TOS, proves that the higher concentration can exert a powerful antioxidant effect, possibly related to a powerful activation of antioxidant cells defenses mechanisms. In our study, Gentamicin administration induces a decrease of antioxidant mechanisms (Table 2, Figure 3). Considerable depletion in renal antioxidant enzymes following Gentamicin administration was also demonstrated by other studies [63,64]. The serum levels of catalase and TAC were significantly influenced by liposomal Curcumin (both doses LCC1 and LCC2) compared with curcumin solution (CC1 and CC2), but no significant differences were obtained when LCC1 and LCC2 administration were compared. This finding suggests that Curcumin’s liposomal formula brings a better efficiency in improving the antioxidant activity in Gentamicin induced nephrotoxicity. Cao et al. demonstrated the beneficial and synergistic effect of Curcumin and metformin in Gentamicin induced nephrotoxicity, protecting against oxidative stress by increasing the antioxidative enzymes’ activity [40]. Furthermore, the combination of curcumin solution with metformin was proved to reduce the inflammatory response induced by Gentamicin, demonstrated by improving the level of the pro-inflammatory cytokine such as TNF-alpha, Il-1 beta and IL-6 [40]. By down-regulating the activity of cleaved Caspase-3 and pro-apoptotic factor Bax and by increasing anti-apoptotic factor Bcl-2 signaling pathways, Curcumin and metformin contributed to apoptosis reduction and improvement of renal function parameters [65]. Our results demonstrated that curcumin efficiency could be augmented by liposomal formula, contributing to improving the oxidative a/antioxidant balance in Gentamicin-induced nephrotoxicity.

### 4.2. Curcumin Influence on MMP-2 and MMP-9 and Histopathological Changes in Nephrotoxicity Induced by Gentamicin

Metalloproteinases are zinc-dependent enzymes contributing to tissues homeostasis and remodeling. Metalloproteinase increased levels could contribute to renal lesions in experimental models of Gentamicin induced nephrotoxicity [10]. Our results also demonstrated a significant increase of MMP-2 and -9 following Gentamicin administration. Cytoarchitecture of the kidney tissue was also influenced by Gentamicin administration (demonstrated by mesangium edema, capillaries narrowing, cellular detritus, and granular protein precipitations), with fewer lesions in curcumin solution and liposomal curcumin-treated groups (Figure 6). Amelioration of metalloproteinases lesional effects by Curcumin was demonstrated by Kim et al. showing the reduction of tissue inhibitor of metalloproteinases 1 (TIMP-1) in cadmium-induced nephrotoxicity in rats [65]. The same study showed that other molecular biomarkers for renal tissue lesions (neutrophil gelatinase-associated lipocalin (NGAL) and netrin-1 were also significantly influenced by curcumin administration as a pretreatment [65]. These protective effects of both curcumin solution and liposomal Curcumin are strongly contributing to lesser histological changes induced by Gentamicin administration by significantly improving the MMP-2 and -9 effects. Liposomal Curcumin was demonstrated to have better histopathological changes than curcumin solution (Figure 6). Histopathological improvement of renal kidney tissue’s cytoarchitecture is possibly related to a decrease of MMP-2 and-9 activity, as have been shown by other studies [40,65]. MMP-2 and -9 increase and TIMP-1 reduction have and important role in the pathophysiology of acute renal dysfunction induced by renal ischemia [20]. The reduction of oxidative stress and improving antioxidant defenses mechanisms showed to be also important in reducing histopathological changes in Gentamicin induced nephrotoxicity [66]. Oxidative stress can induce renal tissue lesions by alteration of cellular membrane integrity and metalloproteinases by the destruction of the extracellular matrix, which in turn results in morphological and functional changes [67].

### 4.3. Renal Function Improvement in Gentamicin Induced Nephrotoxicity by Curcumin Pretreatment

Urea, creatinine, and BUN levels were higher in the Gentamicin treated group, when compared to the control group. Since the estimation of glomerular filtration rate (GFR) is more complex to be routinely assessed, creatinine biomarker is considered the most reliable parameter for assessing renal function in both experimental and clinical practice [68]. Creatinine plasma levels also indicate the structural damage of the kidney [69]. Kidney cytoarchitecture changes by the nephrotoxic effect of Gentamicin are associated with an increase of creatinine, together with blood urea nitrogen (BUN), as an expression of the nitrogen content of the urea (Table 4, Figure 5). Proteins metabolism generates urea, which is the main nitrogenous waste product. Whole urea molecules measurements had the same results (Table 4, Figure 5). These 2 different ways of reporting urea results had the same significantly changes when we compared Gentamicin treated group with the control group and when Curcumin (CC and LCC) groups were compared with Gentamicin treated group) (Table 4, Figure 5). Celik et al. also found significantly increased biomarkers for renal function after Gentamicin administration [70]. Whereas the new nanoformulation of curcumin therapy (liposomal Curcumin) had a better efficiency on renal function biomarkers, we consider that liposomal curcumin pretreatment could significantly contribute to the preservation of renal function in Gentamicin induced nephrotoxicity. The pathophysiological mechanisms implied in these results consist in improvement of oxidative stress/antioxidant balance and consecutive inflammation, improvement of MMP-2 levels and histological aspect, and improvement of biomarkers of renal function. Different mechanisms regarding the beneficial effects of Curcumin on renal function were also reported: reducing pro-inflammatory cytokines (TNF-alpha, IL-6) [71], reducing growth factors synthesis (TGFβ, VEGF, and PDGF), which contribute to renal failure [72], xanthine oxidase blocking with oxidative stress attenuation [72]. Another important mechanism influenced by curcumin administration is represented by reducing NF-κB activation, which is correlated with the reduction of ROS and pro-inflammatory cytokine synthesis, influencing the pathophysiological loop between oxidative stress and overproduction of pro-inflammatory molecules with lesional consequences [73].

### 4.4. Limitations and Future Studies

Although liposomal Curcumin’s use as a protective and therapeutic agent against Gentamicin-induced nephrotoxicity has been successfully investigated, more research is needed to explain the implied molecular mechanisms. Although serum levels were analyzed as markers for gentamicin-induced nephropathy, the assessment of the oxidative stress markers found in the kidney should be another appropriate view of the CC and LCC effectiveness, and this is under investigation in our team. The pharmacological mechanisms of LCC responsible for the alleviation of different types of oxidative stress/antioxidant molecules and MMPs associated with kidney tissue damage also need further investigations. Furthermore, the inside of the renoprotective mechanism of the LCC, as well as changes in gene expression and/or changes in the activity of selected transduction pathways, must be investigated.

## 5. Conclusions

Liposomal Curcumin proved to have beneficial effects in regards to improving renal function in Gentamicin-induced nephrotoxicity. The liposomal Curcumin formulation prevented oxidative stress and augmented antioxidant status, contributing to a better outcome after Gentamicin administration. Histopathological aspects and renal function biomarkers were alleviated by curcumin pretreatment. Significant differences between CC and LCC on serum biomarkers and kidney cytoarchitecture support the conclusion of a higher bioavailability of LCC on kidney function preservation. Considering the liposomal formulation of Curcumin as a promising nutraceutical molecule, our study established a possible link between oxidative stress/antioxidant balance, metalloproteinases synthesis, and histological and renal function changes induced in the nephrotoxicity process. However, the mechanisms able to explain the LCC effects must be further investigated. Due to the various beneficial properties of Curcumin, these results can constitute a starting point for reasonable adaptation to clinical studies.

## Figures and Tables

**Table 1:** Serum levels of oxidative stress intensity.

Group Abbreviation	MDA [nmol/L]	NOx [μmol/L]	TOS [μmol H_2_O_2_ Equiv./L]
C	2.4 (0.2)	27.4 (1.6)	18.5 (1.3)
G	24.1 (1.7)	57.7 (1.6)	81.4 (3.1)
GCC1	22.3 (0.99)	52.8 (1.63)	67.8 (4.16)
GCC2	20.4 (1.6)	46.2 (2)	60 (1.9)
GLCC1	15.5 (1.4)	42 (1.3)	52.7 (1.8)
GLCC2	12.5 (1.4)	38.6 (1.3)	48.8 (1.8)

Values expressed as mean (standard deviation); Abbreviations: MDA, malondialdehyde; No, nitric oxide; TOS, total oxidative status; C, Control; G, Gentamicin; GCC1, Gentamicin and CC1 solution; GCC2, Gentamicin and CC2 solution; GLCC1, Gentamicin and LCC1 solution; GLCC2, Gentamicin and LCC2 solution.

**Table 2:** Serum levels of antioxidant capacity.

Group Abbreviation	Catalase [U/mL]	TAC [mmol Trolox/L]
C	21.3 (2)	1.7 (0.2)
G	9.5 (0.8)	0.7 (0.1)
GCC1	11.1 (1.2)	0.9 (0.07)
GCC2	11.6 (1)	1 (0.1)
GLCC1	14.4 (0.8)	1.2 (0.1)
GLCC2	15.1 (0.8)	1.4 (0.1)

Values expressed as mean (standard deviation); Abbreviations: TAC, total antioxidative capacity; C, Control; G, Gentamicin; GCC1, Gentamicin and CC1 solution; GCC2, Gentamicin and CC2 solution; GLCC1, Gentamicin and LCC1 solution; GLCC2, Gentamicin and LCC2 solution.

**Table 3:** Serum levels of matrix metalloproteinases.

Group Abbreviation	MMP-2 [ng/mL]	MMP-9 [ng/mL]
C	79.3 (2.9)	19.1 (2.5)
G	250.4 (9.3)	41.7 (2.8)
GCC1	212.6 (11.62)	36.7 (2.56)
GCC2	190.6 (11.1)	34.1 (2.5)
GLCC1	155.6 (8.2)	30 (2.2)
GLCC2	115.7 (8.2)	25.3 (2.2)

Values expressed as mean (standard deviation); Abbreviations: MMP-2, matrix metalloproteinases 2; MMP-9, matrix metalloproteinases 9; C, Control; G, Gentamicin; GCC1, Gentamicin and CC1 solution; GCC2, Gentamicin and CC2 solution; GLCC1, Gentamicin and LCC1 solution; GLCC2, Gentamicin and LCC2 solution.

**Table 4:** Serum levels of renal function parameters.

Group Abbreviation	Creatinine [mmol/L]	Urea [mmol/L]	BUN [mg/dL]
C	13.7 (2.2)	14.2 (0.6)	6.6 (0.3)
G	45.3 (1.5)	30.6 (1.4)	14.3 (0.7)
GCC1	39.6 (1.72)	25.9 (1.1)	12.1 (0.51)
GCC2	34.3 (1.4)	23.4 (0.9)	10.9 (0.4)
GLCC1	30.6 (1.9)	20.2 (1.4)	9.4 (0.7)
GLCC2	27.7 (1.9)	17.7 (1.4)	8.2 (0.7)

Values expressed as mean (standard deviation); Abbreviations: BUN, blood urea nitrogen; C, Control; G, Gentamicin; GCC1, Gentamicin and CC1 solution; GCC2, Gentamicin and CC2 solution; GLCC1, Gentamicin and LCC1 solution; GLCC2, Gentamicin and LCC2 solution.

## Data Availability

All relevant data is contained within the article.
